# Sample Preparation for Metabolomic Analysis in Exercise Physiology

**DOI:** 10.3390/biom14121561

**Published:** 2024-12-07

**Authors:** Valeriya I. Nakhod, Tatiana V. Butkova, Kristina A. Malsagova, Denis V. Petrovskiy, Alexander A. Izotov, Kirill S. Nikolsky, Anna L. Kaysheva

**Affiliations:** Institute of Biomedical Chemistry, 109028 Moscow, Russia; vnakhod88@gmail.com (V.I.N.); t.butkova@gmail.com (T.V.B.); petro2017@mail.ru (D.V.P.); izotov.alexander.ibmc@gmail.com (A.A.I.); glucksistemi@gmail.com (K.S.N.); kaysheva1@gmail.com (A.L.K.)

**Keywords:** metabolomics, metabolite, sample preparation, athletes, biosample, storage modes, extraction, omics

## Abstract

Metabolomics investigates final and intermediate metabolic products in cells. Assessment of the human metabolome relies principally on the analysis of blood, urine, saliva, sweat, and feces. Tissue biopsy is employed less frequently. Understanding the metabolite composition of biosamples from athletes can significantly improve our knowledge of molecular processes associated with the efficiency of training and recovery. Such knowledge may also lead to new management opportunities. Successful execution of metabolomic studies requires simultaneous qualitative and quantitative analyses of numerous small biomolecules in samples under test. Unlike genomics and proteomics, which do not allow for direct assessment of enzymatic activity, metabolomics focuses on biochemical phenotypes, providing unique information about health and physiological features. Crucial factors in ensuring the efficacy of metabolomic analysis are the meticulous selection and pre-treatment of samples.

## 1. Introduction

Physical activities often induce profound and extensive modifications in the metabolic processes of numerous organs and tissues. Understanding alterations in many metabolites and metabolic pathways, which are caused by physical activities varying in intensity and duration, is a critical task in sports medicine [1,2,3,4]. According to the Human Metabolome Database, about 220,000 endogenous metabolites and more than 420 biological pathways are known [5]. In contrast to genomics, transcriptomics, and proteomics, metabolomics provides a more direct approach to studying biological activity, offering a reflection of observed biological events. Furthermore, the turnover rate of metabolites is significantly higher than that of proteins and DNA. Metabolites closely interact with genes, proteins, and the external environment. This explains the widespread use of bioanalysis of small molecule composition and content in studies on physiological or pathological states of living systems. The field of sports medicine finds metabolomics particularly relevant, as methods for detecting small molecules were first developed to identify prohibited substances. The current focus of small molecule detection method development lies in monitoring athletes’ health, optimizing training and recovery efficiency, and facilitating dietary adjustments.

**Figure 1:**
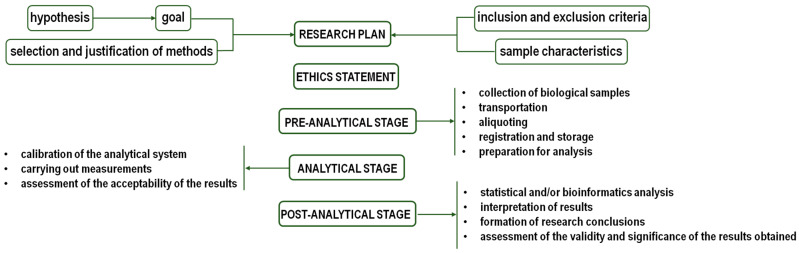
Metabolomics algorithm.

Metabolomics plays a key role in understanding the effects of physical activity on an athlete’s body. Training and competition induce complex changes in metabolic processes in the body. Variations in these changes are influenced by the type and intensity of an activity and individual athlete characteristics. Metabolomics facilitates the identification of metabolic pathways that are either activated or inhibited during training, enabling new strategies for optimizing training, dietary, and rest regimens. Such strategies can enhance athletic performance and maintain a high level of physical condition in athletes. Like any other omics analysis, metabolomics can be represented as three sequential stages: pre-analytical, analytical, and post-analytical (Figure 1).

Contemporary “omics” research adheres to established standards for sample collection, pre-treatment, storage, and transportation, as exemplified by ISO 23118:2021 [6] and other guidelines [7]. A relevant example is a review that delves into pre-analytical variables impacting metabolomic studies of biological fluids. It assesses pre-analytical factors that may introduce artificial data variation, emphasizing their importance in experimental design [8]. Emphasis should be placed on the SPIDIA project, which was dedicated to the creation of standards for mitigating sample variations. This project aimed to ensure that divergent methodologies or environments did not compromise the final results [9]. Unfortunately, the lack of standards for collecting biological material, specifically from professional athletes, hinders the acquisition of accurate, reliable, and reproducible metabolomic analysis results.

Errors at the biological sample collection stage can lead to a distortion of measurement results, affecting the interpretation of data obtained and subsequent solutions. Factors such as time of sampling, the intake of biological supplements, physical activity, and non-compliance with storage and transportation conditions, can significantly alter the metabolite profile. This can distort the true metabolic picture of the body, reflecting instead the consequences of improper handling of biological material or inadequate sample preparation procedures. It can be reasonably argued that the control of biological sample collection and preparation represents the basis for subsequent successful and reliable analysis and accurate conclusions regarding an athlete’s metabolism.

This review summarizes the literature data on the main procedures used in the pre-analytical phase, with a focus on their possible limitations. This summary is intended for researchers in sports medicine who plan and perform metabolomic studies.

## 2. Materials and Methods

We conducted a comprehensive search in online databases, including Google Scholar. The search keywords were “sample preparation” and “metabolomic analysis” and “plasma” or “urine” or “saliva” or “feces” and “athletes”. The study included full-text scientific articles (both reviews and original research) in English, published in peer-reviewed journals indexed in Scopus or Web of Science. We excluded duplicate articles, irrelevant studies, and those lacking information on sample collection and pre-treatment procedures.

## 3. Results

### 3.1. Factors Affecting Metabolomic Study Results

#### 3.1.1. Blood

The metabolomic composition of blood is a strictly controlled homeostatic system, but various physiological conditions and exogenous factors cause changes in the composition and content of metabolites. Beyond the possible impact of the pre-analytical phase [10], the composition of the blood metabolome is significantly influenced by numerous internal and external factors. These include, but are not limited to, circadian and physiological rhythms [11], diet [12], physical exercises [13], medications [14], and other variables [15]. Table 1 summarizes the most important recommendations for sample collection.

Gender differences, age, and body mass index are crucial factors in metabolomic studies. Another significant factor is nutrition. Nutrimetabolomics is a field of research that studies the relationship between diet and health [30]. Abstinence from food is recommended before collecting biological samples because the blood metabolite profile undergoes dynamic changes within a few hours after eating [24,31]. A substantial portion of the blood metabolome is sensitive to food intake. The postprandial metabolome is affected by food composition [31]. For instance, notable alterations in essential amino acid and acylcarnitine concentrations are observed 3 and 5 h postprandially [24]. According to medical examination standards, as well as established practices in metabolic studies [23,24], a 12 h fast is recommended for assessing metabolic function. The time of day at which blood samples are collected significantly influences the resulting metabolite profile [27]. For example, blood levels of lipids, branched-chain amino acids, and lactate are closely associated with biorhythms [32]. The light–darkness cycle, sleep and wakefulness patterns, composition of the last meal, and other factors should be taken into account in metabolomic studies. The effects of external factors on metabolomics results should be minimized by collecting all samples at the same time point and under the same conditions, providing more homogeneous and comparable data. Selecting a specific time of day for sample collection, such as early morning, may yield more stable metabolite concentrations due to the absence of postprandial or post-exercise fluctuations. It is also essential to take into account the influence of food on the metabolic profile. Samples should be collected before eating (under fasting conditions) to avoid the effects of various food components on the composition and content of metabolites in analyzed samples. Some studies require postprandial metabolomics data to be analyzed. Such time-resolved analysis can enhance the comprehension of the metabolic processes of a participant or a group of participants, identify candidate biomarkers of health and unhealthiness, and facilitate the creation of dietary and nutritional recommendations [33]. It is recommended that time points for analysis be selected within the first hour to 6 h after a meal [34,35,36,37]. Physical exercise and stress are also important factors affecting metabolism. Exercise training significantly alters the composition of metabolites in athletes’ blood, with the magnitude of change being primarily influenced by the intensity and duration of the training regimen [38]. Exercise metabolomics serves as a valuable resource for generating data that can be utilized in developing approaches to improve human performance, promoting exercise as a countermeasure against chronic diseases and advancing research in sports science [38]. Comparative investigations of metabolite profiles enable the elucidation of biological processes underlying physiological adaptations to exercise, including enhanced muscle strength or aerobic metabolic capacity and their impact on health [3,38,39,40]. Post-exercise metabolic changes in the body are considerably affected by factors such as the intensity, duration, and frequency of the exercise performed [2,3,38,41]. Investigations into the effects of varying loading conditions and loading durations on changes in the metabolite profile of blood commonly incorporate a sequence of time points: pre-loading (0 h), post-loading (30 min to 2 h), with subsequent evaluations at 24 h [29,42,43,44,45]. In select studies, observations are sustained for an extended duration, reaching a maximum of 14–21 days. Exercise-induced alterations in various biochemical markers, including lactate, amino acids, and acylcarnitines, have been documented, with corresponding decreases observed in fatty acid levels [24,29,46]. It is also important to note that lifestyle, especially unhealthy habits, can affect the metabolic profile [47]. Consequently, evaluating the lifestyles of study participants through questionnaire data (Table S1) can be an effective approach to research planning. Table 2 presents some metabolites found in plasma and serum.

The data in Table 2 are partially explained by the fact that blood coagulation can lead to changes in concentrations of certain metabolites due to the removal of proteins and the release of substances from blood cells, e.g., hypoxanthine, xanthine, and amino acids [53,54], as well as enzymatic reactions [53,55]. Thus, coagulation conditions for the production of serum should be strictly controlled to minimize enzymatic reactions and metabolic changes, so particular attention should be paid to reproducible standard operational procedures to ensure low variability among samples. However, the use of plasma has a number of advantages, such as faster and simpler processing and high reproducibility compared with serum, due to the lack of coagulation. When choosing between serum and plasma, it is necessary to take into account these factors and select the most appropriate method for a particular study [48].

Blood plasma and serum samples can also contain various exogenous components. Rubber plugs, surfactants, and barrier gels used in test tubes can be sources of contamination [55]. Plastic polymers, plasticizers, and anti-slip agents present in plastic consumables can mask metabolites in analysis [56]. In addition, plastic consumables contain low molecular weight compounds similar to endogenous metabolites, such as free fatty acids, which complicate their analysis [57].

The metabolomic composition of serum also depends on the type of test tube used for collection. The utilization of polymer gel tubes results in alterations in the concentrations of amino acids (alanine, proline, and threonine), glycerol, monopalmitin, monostearin, aconitic acid, and lactic acid [58]. Coagulation-promoting silicate-coated test tubes have been shown to influence serum metabolite composition, resulting in a higher abundance of the phenylalanine–phenylalanine dipeptide [55].

Anticoagulants used in blood sample preparation can influence the metabolomic profile of plasma. Contamination of plasma samples with anticoagulant cations (lithium, sodium, or potassium) significantly affects the results of mass spectrometric metabolomic analysis, causing ionization suppression or enhancement of adduct components analyzed [59]. Lithium ions from heparin can increase the efficiency of ionization of phospholipids and triglycerides, among other metabolites [60]. Anticoagulants were also demonstrated to impact the levels of amino acids, carbon acids, and polyols [61]. Test tubes with ethylenediaminetetraacetic acid (EDTA) should not be used for analyzing polar metabolites. The use of test tubes with sodium citrate is known to complicate the analysis of citric acid and its derivatives. Electrospray ionization, in both positive and negative ion modes, identified a greater number of metabolites in plasma collected in heparin tubes relative to samples collected in EDTA, citrate, or oxalate tubes [61]. Also, plasma collected in EDTA test tubes had a richer lipid profile (glycerophospholipids, sphingolipids, diacylglycerols, triacylglycerols, cholesterol esters, and acylcarnitines) and increased amino acid contents (aspartate, histidine, and glutamine) than that collected in citrate test tubes [62]. Anticoagulants can also affect the efficiency of extraction and derivatization processes.

Another common and important factor affecting metabolism and outcomes in research is the intake of medications and supplements [4]. It is imperative that the questionnaire include information regarding drug therapy to ensure that the metabolic effects of medications are not mistakenly attributed to the experimental outcomes. Dietary supplements, encompassing those containing polyunsaturated fatty acids, multivitamin preparations, amino acid and protein shakes, among others, also influence metabolic processes. Since nutritional supplements are not drugs and are not considered to affect the study results, they are less frequently mentioned in the questionnaire. The influence of these supplements on metabolomic study results should not be disregarded, as this could potentially lead to misinterpretations of the data obtained. As a result, it is advisable to include a section in the survey questionnaire that addresses nutritional supplements and medications.

The sampling process also involves other important aspects. To ensure consistency and prevent systematic error, all participants should be punctured at the same site throughout the study [63]. The system used to collect blood can also significantly affect the final composition of blood samples. The vacuum system (e.g., Vacutainer test tubes) is preferable because it provides safe blood collection in constant volume. In turn, aspiration systems (e.g., Monovettes) enable the control of vacuum, reducing the risk of hemolysis (i.e., erythrocyte lysis). Blood collection tubes should be filled with the same sample volume to ensure repeatable concentrations of additives and contaminants for all analyzed biological samples (e.g., anticoagulants, residual polymer coating, chemicals, etc.). The differences in the metabolite composition of samples can be due to the use of alcohol wipes containing various surfactants, detergents, antimicrobial agents, and stabilizers for skin sterilization before blood collection. These agents can further be detected in blood samples [64].

Hemolysis is a prominent risk factor associated with blood sampling [65,66,67] and a prevalent cause of pre-analytical errors. In addition to excessive biosample aspiration, other contributing factors to hemolysis include excessive tube shaking, disregard for optimal temperature conditions during transportation, high centrifugation speeds, and inadequate ambient temperature. Hemolysis causes the release of intracellular compounds such as metabolites and enzymes that can significantly change the metabolite profile of the blood sample. Erythrocyte lysis leads to an elevation in the concentrations of intracellular metabolites, specifically tryptophan and cell membrane lipids.

Hemolytic and non-hemolytic samples are easily distinguished by color because free hemoglobin changes the color of serum or plasma from pale yellow to bright red. This color shift, however, is not evident in samples with low hemolysis, especially when the donor exhibits elevated bilirubin levels. Along with hemolysis, hyperbilirubinemia and lipemia are also common pre-analytical problems that affect the results of routine clinical tests [68]. High lipid or bilirubin concentrations, which can be problematic for routine clinical visual analyses, do not typically hinder metabolomic analysis using liquid chromatography and mass spectrometry.

The choice between serum and plasma as the matrix for analysis is subject to ongoing debate, as these biofluids can yield divergent values for various metabolites. Nonetheless, response patterns to a particular treatment or condition generally exhibit consistency, regardless of whether serum or plasma is employed. It should be noted that while absolute values may vary between these matrices due to differences in processing and components, the relative changes and trends in response to interventions generally align, providing evidence for the robustness of the findings across both sample types.

The optimal timing for material sampling can vary significantly depending on the specific parameters under consideration [69]. Table 3 provides a comprehensive review of the different collection and preparation methods used for plasma and serum samples in studies examining the association between physical activity and metabolite profiles.

The data presented in Table 3 indicate that EDTA-containing tubes are the preferred choice for plasma collection in metabolomic studies. However, a notable disparity exists among researchers regarding the precise conditions used during sample preparation, specifically with respect to centrifugation parameters (speed and duration). The researchers, however, do not cite any sources that recommend these specific conditions, including Standard Operating Procedures (SOPs) and other standards.

#### 3.1.2. Urine

Urine is a preferred biofluid for metabolomics studies owing to the ease and non-invasive nature of obtaining large volumes of biomaterial. The metabolic profile of urine, similar to other bodily fluids, is subject to constant change. In sports medicine research, it is crucial to consider the influential factors (Table 1) that determine the composition and content of endogenous metabolites in urine. Dietary and exercise regimens significantly influence alterations in urinary metabolites [88,89,90]. Determining reliable indicators of dietary quality and bodily health is a significant objective within the discipline of sports nutritionology [89]. The duration of the study is contingent upon the specific task being undertaken. Prior to implementing dietary modifications, it is crucial to ascertain the metabolic profile of the body at baseline. Moreover, the selection of sampling time points can be structured as follows: within a few hours [91,92,93], a weekly basis, a biweekly basis, a monthly basis, and thereafter, at intervals longer than a year [89,94]. Exercise, load, duration, and frequency are factors that impact the composition of metabolites in urine. Thus, aerobic exercise affects metabolites in urine through the purine pathway, the tricarboxylic acid cycle, tryptophan, carnitine, cortisol, and androgen metabolism, and amino acid oxidation [95,96]. Study time points may include both baseline measurements and post-load measurements collected within a 24–48 h timeframe [95,96,97] or even extended to several days. Numerous sample collection methods, including spot, timed, and twenty-four-hour sampling, have been documented for the bioanalysis of urine (Table 4).

Collection modes significantly affect the metabolic composition of these urine samples and, therefore, should be taken into account when developing a study design [105]. A mid-stream specimen of urine is traditionally preferable for random spot sampling. This approach minimizes contamination by bacteria, cells, and particles from the genital and urethral mucosa. However, there may be minor differences in the composition of metabolites between early- and mid-stream specimens of urine [106]. It is recommended that the collection be conducted early in the morning, immediately following a night’s sleep, prior to breakfast and any physical exertion. In this case, urine is more concentrated compared to samples collected at another time during the day. It is the morning urine collection that is widely used in nutrimetabolomics and exposure assessment [107]. In contrast, the analysis of second-morning urine minimizes the influence of dietary factors on metabolic urine profiles, facilitating the detection of clinical biomarkers [108]. An alternative approach involves collecting urine samples at various time points to investigate transient metabolic patterns. Such studies may encompass, for instance, the kinetics of ingested substances (e.g., food products, nutrients, or xenobiotics), metabolic alterations associated with the circadian rhythm, or other time-dependent variations. For this purpose, the control urine sample is usually collected at the beginning of the study and then at different time intervals (for example, 0–2, 2–6, and 6–12 h). All urine samples collected during each interval are stored in the refrigerator and then pooled. The third sampling method involves collecting all urine produced throughout the day and combining it into a single 24 h specimen. Such an approach reduces the variability in metabolite composition, which is typical of shorter sampling intervals, and provides a general picture of the excretion. However, 24 h urine collection is burdensome and most prone to errors. This urine collection method requires proper storage of urine by study participants at their homes before sending it to the research center. Thus, although 24 h sample selection may be preferable in terms of minimal metabolite concentration variations, spot sampling is also widely used in metabolomics due to its ease of use in clinical practice.

Empty polypropylene containers are usually used to collect urine. However, some researchers recommend using surfactant additives to increase the solubility of lipophilic metabolites that can adsorb on the container walls [109]. As in blood sample collection, surfactants significantly affect the subsequent metabolite analysis using mass spectrometry. As a result, their utilization has not become widespread. Urine samples collected over a specific interval or for a 24 h period should be refrigerated until pooling and storage. Some preservatives can be added to avoid bacterial growth and resulting metabolomic alterations. For example, adding hydrochloric acid to the sample preserves some urine metabolites, e.g., citric acid [110]. Unfortunately, there is no gold standard for the complete preservation of urine metabolome [111]. Therefore, the reliability of the results obtained through untargeted metabolomics could be considered uncertain.

#### 3.1.3. Saliva

The shifts in saliva composition during and subsequent to exercise demonstrate the dual nature of physical activity—exhibiting both its beneficial outcomes and potential hazards—and bolster the notion of saliva as a viable indicator of an individual’s health. Exercise-induced changes, hormone adaptation, lactate accumulation, and shifts in immunological markers can be assessed non-invasively through saliva analysis [112]. Salivary gland activity is influenced by both parasympathetic cholinergic and sympathetic adrenergic nerve stimulation. Parasympathetic stimulation enhances regional blood flow and salivary secretion, characterized by a reduced concentration of organic and inorganic constituents. Sympathetic stimulation elicits the secretion of a small volume of saliva with elevated potassium and protein concentrations [113]. The analysis of hormonal responses during exercise can provide valuable information regarding training stress, adaptation, dehydration, and performance [114].

Exercise has been linked to an increase in salivary and protein secretion, including enzymes like amylase and lysozyme, as well as the protein MUC5B [115]. A decrease in saliva production can alter the levels of substances, like metabolites, found in saliva. Research suggests that intense exercise results in a reduction in IgA by over 30% [116].

Prolonged, intense exercise can lead to overtraining syndrome, resulting in a decline in athletic performance and cortisol levels. Consistent saliva sample collection, aligned with the circadian rhythm, is crucial for measuring cortisol concentrations and understanding exercise stress. This information is essential for understanding and managing overtraining syndrome in athletes [117,118,119].

The choice of sampling method is crucial, as the analysis of metabolites utilizes different saliva types: unstimulated whole saliva and whole saliva stimulated by citric acid. The conditions for sampling and processing saliva can significantly affect its composition and study results. For example, the composition of stimulated saliva differs from that of unstimulated saliva. Chewing-stimulated saliva is considered an adequate alternative to unstimulated saliva for microbiome studies [120]. Therefore, the plan for saliva sampling depends on the hypothesis and research design.

Saliva collection is a painless, inexpensive, and simple sampling procedure with no risk of infection for study participants. The multicomponent nature of saliva underscores the importance of standardized collection and storage procedures.

Table 5 details the saliva collection procedures employed in studies that analyze the metabolite profile of saliva during exercise.

The pre-analytical stage necessitates standardized protocols for sample collection and storage. The general stages of preliminary preparation of saliva samples are centrifugation and storage at low temperatures. Centrifugation is an important step for removing the contents of the cells. Repeated freezing and thawing of saliva samples do not affect the profile of metabolite content [125].

Individual features and daily changes in the saliva composition can affect the results of analytical measurements to a greater extent compared with differences in storage and handling conditions. In order to ensure sample stability, microbial growth inhibitors, such as sodium azide (NaN_3_), can be employed. In general, saliva samples are quite stable. Samples can be stored at a temperature of −20 °C for up to 4 weeks without adverse effects on tests [126].

#### 3.1.4. Feces

Unlike urine or plasma, feces are not a homogeneous sample. A stool sample usually contains various areas or topographical locations that can vary greatly in metabolite composition. One of the strategies for solving this problem is to collect several fractions from different topographical locations and pool them into the final sample that is then processed to extract metabolites.

Human feces contain 75% water, on average, but this indicator varies depending on the diet, fiber consumption, and hydration [127]. In this regard, the content of metabolites varies on different days or even within a day. Two main methods used to minimize variability in water content are based on the separation of water and solids in stool samples. In the first case, the water phase of feces (usually called fecal water) is used for metabolomic analysis. This means that the metabolites remaining in the solid phase are not analyzed. The second method is to remove the water fraction by lyophilization and analyze the metabolites remaining in the solid fraction. Like other pre-analytical procedures, lyophilization also affects the content of certain metabolites.

Evidence indicates a strong connection between gut microbiota and the overall health of athletes [128]. Furthermore, it is important to note that the metabolic activity of the predominant microbial community in the large intestine of athletes could have an effect on their athletic performance [129]. The diversity and composition of the gut microbiota in professional athletes undergo significant changes in response to extreme physical activity, distinct from those observed in individuals with less active lifestyles [129,130,131]. Athletes exhibit proportionally elevated levels of the metabolite trimethylaminoxide, a compound known to induce oxidative stress. An elevation in the levels of various metabolites associated with muscle metabolism, including creatine, 3-methylhistidine, L-valine, and carnitine, reflecting the body’s metabolic state, has been observed [98]. Enhanced production of short-chain fatty acids, resulting in increased energy intake, was observed in healthy athletes, consequently contributing to overall metabolic efficiency [132,133,134].

Comparative analysis of metabolites found in fecal water and lyophilized feces revealed that lyophilized samples contained a wider range of metabolite classes [135].

It is recommended to carefully plan the design of analysis of fecal metabolite composition and first conduct a test study on several samples. If possible, preliminary sample preparation protocols should be compared. After optimization, the most appropriate study protocol should be used.

The following are fundamental guidelines for sample collection, which must be carefully observed.

- Prompt specimen handling is crucial to mitigate sample degradation. Samples should be maintained at 4 °C and, upon completion of lyophilization, if applicable, promptly frozen at −80 °C.
- The partial biological activity of the sample necessitates a rapid freezing process. Warmer conditions stimulate the growth of living microorganisms in the sample, leading to potential biases in metabolomic studies.
- Experiments must be meticulously planned, taking into account factors like sampling time, patient fasting status, and dietary intake, which can profoundly impact metabolism and the measured sample values.
- One should acquire representative fractions of stool sample material from diverse topographical locations within the sample for subsequent processing.
- To mitigate biological variability arising from differing water content, it is advisable to utilize lyophilization to remove water when employing target metabolomics kits.

It is important to note that the collection methods employed for fecal samples vary substantially across studies. While some research teams regulate the diet and fluid intake of participants, others fail to elucidate these details (Table 6).

### 3.2. Sample Preparation Features

#### 3.2.1. Features of Plasma and Serum Preparation

Sample preprocessing constitutes the final stage of pre-analytical procedures. Usually, this stage is strictly standardized and less prone to errors.

The main stages of blood sample preprocessing for metabolomics include deproteinization and extraction (Table 7).

Usually, organic solvents, ultrafiltration, and solid-phase extraction (SPE) are used to precipitate proteins [142]. In particular, organic solvents, such as acetonitrile, methanol, chloroform, etc., can be used for high-performance extraction of, e.g., polar metabolites [143,144] and lipids [145]. The strategy for using methyl tert-butyl ether, methanol, and water to extract metabolites from tissues enables simultaneous effective extraction of polar and non-polar metabolites. This method is also suitable for blood sample preparation [145]. Each of these procedures is effective but has limitations due to possible loss of one or more metabolite subclasses.

Critical variables for solvent- and SPE-based extraction are the type of solvent, pH, and temperature. In SPE, the solid-phase material determines the content of which metabolite classes will be reduced or even lost. Nevertheless, SPE is applicable for profiling biosamples with low metabolite contents through their concentration.

The simplest preprocessing of the sample for mass spectrometric metabolomic analysis guarantees high repeatability. Deproteinization should include the addition of an organic solvent, followed by centrifugation under strictly standardized conditions because this procedure is simple, widespread, and effective [146]. Another essential procedure is the thawing of biological fluids in ice water, but not at room temperature. Samples should be stored chilled at all stages, which guarantees their high quality.

#### 3.2.2. Features of Urine Preparation

In the study of human biological fluids, it is important to preserve the initial composition of the metabolome [147]. In addition to the direct injection of the urine sample into the LC-MS/MS system (combination of liquid chromatography and tandem mass spectrometry) [148], preliminary filtration can be used to remove suspended particles. The procedure is usually performed using cellulose filters with a pore size of 0.2–0.45 μm [105]. The use of special membranes with a pore size of 3–30 kDa was reported in a study of hydrophilic metabolites [149]. Also, filtration prevents bacterial growth upon storage [150]. Urea, the final product of amino acid and nitrogen metabolism in humans, is a widespread metabolite found in urine; it can undergo derivatization, generating multiple peaks in mass spectra (Table 8).

Some metabolites displaying a chromatographic retention time similar to that of urea may not be detected in mass spectrometric analysis. In addition, a large amount of urea can induce other problems, including excessive use of the derivatization reagent, column overloading, peak distortions, matrix effects, and reduced MS column lifetime [151]. A universal approach to solving these problems is preprocessing urine samples with urease. Removing urea provides higher repeatability and reproducibility of data compared to unprocessed samples [152]. There are no reports about the optimal amount of urease, but preprocessing by urease is supposed to be particularly effective if the urine sample volume is 25 μL or higher [153]. However, commercial urease enzymes may contain contaminants [154].

Urine is considered an optimal biofluid for metabolomics because it contains a wide variety of metabolites at relatively high concentrations. However, there is significant variability in these metabolites due to various influencing factors, which can hinder the reliability of the data obtained. Numerous endogenous compounds undergo biotransformation, specifically through conjugation, which is the second phase of metabolism. As a result, many of these compounds are excreted in the form of unconjugated species, as well as glucuronides or sulfates. Additionally, conjugation with other compounds, such as N-acetylcysteine or amino acids taurine and glycine, is commonly observed in volatile organic compounds [155] or bile acids [156]. The relative amount of different conjugates depends on several factors, such as enzyme polymorphism or diet. Therefore, the influence of metabolite on the status under study can remain hidden due to intergroup variability in conjugation reactions. Hydrolysis prior to LC-MS analysis can minimize this effect. In untargeted metabolomic studies, preserving the sample intact is important to preserve metabolites in their initial forms [157]. Consequently, hydrolysis finds primary application in targeted investigations designed for the elucidation of metabolic pathways where phase II metabolism holds significant importance. Researchers can employ both enzymatic and chemical hydrolysis methods. When selecting a hydrolysis method, one should consider factors such as the efficiency of hydrolysis and the possibility of artifacts that may alter the response in LC-MS/MS. For example, enzymatic hydrolysis is preferable for analyzing steroids [158], while chemical hydrolysis is considered more effective for detecting catecholamines in urine [159].

An additional parameter that significantly affects the assessment of urine metabolism is a wide range of concentrations of potential biomarkers. Some metabolites in urine are present in micrograms per milliliter. These highly represented metabolites can be detected using targeted and untargeted strategies. The main problem associated with their quantification by LC-MS/MS is related to a limited range of linear dependence for high concentrations. This limiting factor is eliminated by diluting the sample, usually with a dilution coefficient of up to 100 [148]. Quantitative data obtained for the main metabolomic components in samples with low dilution should be interpreted carefully. Alternatively, the concentration of metabolites in urine is often in the nanogram per milliliter range or lower. Despite their low concentrations, their monitoring can be crucial for evaluating metabolic pathways. Most of these metabolites are not accounted for in untargeted approaches due to limited instrumental sensitivity that can be increased by using a preconcentration stage. In this case, several strategies can be exploited. The most popular of these are SPE and liquid–liquid extraction [160]. Given the substantial volume available, preconcentration is typically performed at a 10- to 50-fold level. The implementation of these sample preparation stages increases the overall sensitivity of the analysis [161].

One of the well-known advantages of LC-MS/MS procedures is the lack of a derivatization procedure. However, derivatization increases the limits of quantification of targeted metabolites. In addition, special reactions of metabolite derivatization often increase the efficiency of purification, extraction, and chromatography [162].

The use of isotopes in the preliminary preparation of the urine sample increases the number of metabolites under study. For example, dansylation enables one to label untargeted metabolites containing primary amino, secondary amino, or phenolic hydroxyl groups [163]. In addition, labeling with dansyl chloride upon derivatization improves quantitative parameters in untargeted metabolomics approaches [164].

#### 3.2.3. Features of Saliva Preparation

There are different protocols for the preliminary preparation of saliva. Usually, the sequence of operating procedures depends on the selected analytical platform, method, and study object. The initial step involves pre-centrifugation of the sample to remove macromolecules and cellular elements [165,166,167].

Hydrolysis (both alkaline and acidic) of saliva is a suitable strategy to increase the number of metabolites under study [168]. In addition, the application of ultrasound in hydrolysis increases the number of metabolites to be identified (Table 9).

Compared to other biological fluids, the number of biological molecules in saliva is relatively small, which may be associated with low density (water comprises 99% of saliva). Therefore, it is preferable to perform preconcentration. The use of a concentrated saliva hydrolyzate treated with ultrasound provides an increase in identified objects [168], which confirms topicality of the concentration procedure as a stage preceding the chromatographic analysis.

Organic compounds, such as methanol or acetonitrile, are used to extract metabolites [166,168,169]. In this case, the 1:2 (*v*/*v*) saliva-to-acetonitrile ratio is most suitable for preparing saliva samples in terms of the number of extracted metabolites and repeatability.

#### 3.2.4. Features of Feces Preparation

Preprocessing of fecal samples commonly entails dilution with an organic solvent (e.g., methanol), centrifugation to remove proteins and sediments, and filtration to avoid contamination of the mass spectrometric system (Table 10).

In tandem analysis of metabolites using a quadrupole time-of-flight mass spectrometer, they are extracted using methanol in a ratio of 3 mL/g, followed by centrifugation and filtration through a 0.2 μm membrane [170].

Apart from methanol, metabolites can be extracted with chloroform. Acetonitrile can be used to precipitate proteins [135].

### 3.3. Features of Biological Material Storage

General requirements for storage conditions for biomaterial samples until their shipping to the laboratory are as follows [171]:

- to follow the storage conditions of biosamples until their delivery to the laboratory (Table 11);
- to arrange a storage area for biosamples in departments of a medical institution until their shipping to the laboratory (Table 12);
- to label the biosample storage area with the “Biohazard” sign;
- to store biosamples in closable containers labeled with the “Biohazard” sign;
- to provide regular disinfection of the biosample storage area and containers;
- to put the accompanying documents (referral to study forms) in a plastic bag (plastic pocket) to exclude contact with biomaterial.

Storage of plasma at room temperature for 16 h leads to minor changes (23%) in its metabolite composition [173]. Plasma stored at a temperature of 37 °C for 1 h is very unstable; however, storage of plasma or serum at 37 °C is quite unusual. On the contrary, long-term exposure to low temperatures causes fewer metabolite alterations. Upon storing plasma with EDTA at 4 °C for 16 h, only 30 out of 262 metabolites undergo minor changes [174].

Plasma storage at a temperature of −20 °C leads to significant alterations in some metabolites, in particular glucose and proline [175]. On the contrary, according to the results of a clinical, biochemical study, 17 common clinical serum parameters, including metabolites, such as bilirubin, uric acid, cholesterol, creatinine, and triglycerides, are relatively stable upon storage at a temperature of −20 °C for three months [176]. Storage of plasma or serum at −70 °C and below is preferred [177]. However, there may be changes in the metabolomic composition of samples over time [173]. Therefore, metabolome stability during long-term storage remains an open question.

A research study conducted by Wagner-Golbs et al. investigated the impact of prolonged storage on the metabolome. EDTA plasma samples, stored for up to sixteen years, were analyzed using gas and liquid chromatography-based metabolomics coupled with tandem mass spectrometry. During the initial seven-year storage period, a variation in the concentration of 2% of the 231 metabolites was detected. However, at longer storage times, the changes in composition and metabolite content were ~26%. The most notable changes were evident in compound classes, including complex lipids, fatty acids, molecules involved in energy metabolism, and amino acids. One should note that the human blood plasma metabolome is quite stable to prolonged storage at −80 °C for up to seven years, with longer-term storage, however, resulting in significant changes. Therefore, for retrospective research on EDTA plasma samples, analysis should be performed during the first seven years of storage [178].

In another study [179], the variability of the human serum metabolome was analyzed during prolonged incubation of blood (6 h) and prolonged storage of serum at room temperature (24 h). It was demonstrated that prolonged incubation of blood leads to a statistically significant increase of 20% and a decrease of 4% in 225 serum metabolites studied. The storage of serum for 24 h resulted in a 14% and 7% increase and decrease in metabolite composition, respectively. The largest percentage changes were observed in amino acids and nucleic bases across both conditions, while lipids remained unchanged. A notable increase was observed in the levels of taurine and O-phosphoethanolamine, rising by 1.8 and 2.9 times, respectively, following 6 h of blood incubation. The authors advocate for the utilization of EDTA plasma as a metabolomic matrix based on their findings, as the two compounds demonstrated enhanced stability within this medium. Prolonged incubation of blood (6 h) and serum storage (24 h) at room temperature generally lead to alterations in the human serum metabolome. Consequently, implementing stringent protocols for auditing sample handling is imperative in both clinical and omics research to ensure the attainment of reliable results and reproducibility.

## 4. Conclusions

The field of sports laboratory diagnostics, a continually evolving branch of sports medicine, is primarily concerned with obtaining objective data regarding an athlete’s functional state and health. The timely monitoring of the athlete’s metabolic status during physical activity is critical for preventing diseases and injuries, as well as for evaluating the effectiveness of training and recovery processes in athletes. The achievement of reliable and reproducible comparable outcomes necessitates the standardization of protocols for the preparation of biological samples.

Physical activity constitutes a crucial pre-analytical variable, exerting a considerable influence on the metabolic processes occurring within the human body. Shifts in the quantities and qualities of components within metabolic pathways are associated with corresponding changes in clinical and laboratory indicators.

Therefore, the standardization of the pre-analytical phase is essential for reducing variability among biological samples and preserving the integrity of their metabolite composition. This process involves unifying sample collection, storage, and preparation protocols while adhering to standardized analytical methodologies. Standardization decreases the variation in results while promoting their replicability, which is essential for comparing data across different laboratories and studies.

## Figures and Tables

**Table 1:** Algorithm for evaluating factors affecting metabolomics results.

Assessed factor	Recommendations	Metabolites	Ref.
Gender	Maintain equal gender distribution	Lipids, orthophosphate, α-tocopherol, creatinine, dehydroepiandrostenedione sulfate, and cholesterol	[15,16,17,18]
Age	Maintain age group uniformity across study participants	Amino acids, isocitrate, succinate, malate, lactate, etc.	[18,19]
BMI	Maintain BMI value uniformity across study participants	Branched-chain amino acids, lipids, and steroids	[20,21,22,23]
Diet	Adhere to a 12 h fast (usually overnight)	Essential amino acids and acylcarnitines, triglycerides, and homocysteine	[24,25]
Circadian rhythm	Collect samples at the same time (usually in the morning)	Lipids, branched-chain amino acids, lactate, bilirubin, and cortisol	[26,27,28]
Physical activity/stress	Avoid unusual physical activity and stress before blood collection (survey of study participants)	Lactate, free fatty acids, glucose, amino acids and acylcarnitines, uric acid, creatinine, etc.	[15,24,29]
Medications and/or nutritional supplements	Avoid taking medications and nutritional supplements (24 h or more)	–	-

BMI—body mass index.

**Table 2:** Contents of some compounds in serum and plasma.

Metabolite	Serum vs. Plasma	Ref.
Amino acids		
Arginine	↑	[48,49,50]
Serine	↑	
Phenylalanine	↑	
Glycine	↑	
Glutamate	↑	
Cystine	↑	
Phenylalanine	↑	
Ornithine	↑	
Methionine	↑	
Proline	↑	
Isoleucine	↑	
Valine	↑	
Tryptophan	↑	
Nucleotides		
Hypoxanthine	↑	[50]
Xanthine	↑	
Other metabolites		
Glycerol 3-phosphate	↑	[50,51,52]
Hydroxybutyrate	↑	
Ribose	↑	
Glucose	↑	
Pyruvate	↓	
Citrate	↓	
Fumarate	↓	
Glycerate	↓	
Urates	↓	
Xylitol	↓	

↑ Indicates compounds whose levels in plasma are higher than in serum; ↓ indicates compounds whose levels in plasma are lower than in serum.

**Table 3:** Collection and pre-treatment of blood samples for metabolite analysis.

Study Purpose	Blood	Tube Type	Conditions of Collection	Conditions of Sample Preparation and Storage	Explanation	Ref.
To analyze the effect of long-term physical activity on serum metabolome	Serum	–	12 h-fast	–	–	[70]
To analyze aerobic fitness level and metabolic profile	Plasma	EDTA tubes and lithium heparin tubes	12 h-fast	Centrifugation at 1800× *g* for 15 min at 4 °C; Storage of 500 mL aliquots at −80 °C	–	[71]
To analyze the relationship between blood metabolic profiles and moderate- or high-power endurance sports	Plasma	–	12 h-fast	Extraction of metabolites no later than six hours after collection; Storage of samples at −80 °C	Blood samples were collected in the morning between 8:30 and 10:30 *.	[72]
To analyze the relationship between physical activity level and metabolic profile	Plasma	EDTA tubes	–	–	–	[73]
To analyze the association of diet, physical activity, cardiorespiratory fitness, and obesity with serum metabolome	Serum	Monovette tubes with coagulation inhibitor	12 h-fast	Storage of serum samples stored at −80 °C	–	[74]
To analyze the metabolic response to acute hypoxic exercise and recovery	Serum	–	–	Collection within 36 h followed by delivery to anti-doping laboratories; Centrifugation; Storage at −20 °C	–	[75]
To analyze the relationship between physical activity and the characteristics of the metabolic composition of the blood of adolescent	Blood	–	Blood samples were taken on an empty stomach.	–	Blood was collected during fasting, mostly at the same time of day *.	[76]
To analyze the relationship between physical activity and plasma metabolite levels	Plasma	–	–	Sample collection independently by the study participants; Centrifugation; Storage at −130 °C in liquid nitrogen	Participants were sent a blood collection kit to be returned within 24 h of blood collection.	[77]
To analyze the metabolic response to acute hypoxic exercise and recovery	Serum	SST tubes	Samples were collected after 30 min of rest under hypoxia; Samples were obtained 3 h after training	Blood clotting at room temperature; Centrifugation at 3500× *g* for 10 min; Storage at −70 °C	Load tests were performed between 08:00 and 10:00.	[43]
To analyze the effect of long-term physical activity on metabolic profile	Plasma	EDTA tubes	Samples were collected before and immediately after completing the distance	Centrifugation at 2000× *g* for 10 min at 4 °C; Storage at −80 °C	Food and drinks were provided without restrictions. Participants were asked to refrain from physical exercise and alcohol consumption for 24 h before the start of the test.	[78]
To analyze the effect of long-term physical activity on metabolic profile	Serum	Standard 10 mL tubes	Individuals were required to be hydrated and fasted for at least 2 h at baseline venesection	Blood clotting at room temperature (30 min); Centrifugation at 3000× *g* for 10 min; Storage at −80 °C	Samples were collected the day preceding the race (between 10 a.m. and 6 p.m.) **.	[79]
To analyze the metabolomic response after resistance exercise	Serum	Vacutainer tubes	Blood sampling before and after exercise	Blood clotting at room temperature (30 min); Centrifugation at 1000× *g* for 15 min; Storage at −80 °C	–	[80]
To analyze the changes in plasma metabolome in response to cycling exercise	Plasma	Lithium heparin tubes	10 h-fast; Sampling 30 min before training, 10 min while cycling, and 20 min after training	Centrifugation at 1800× *g* for 10 min; Storage at −80 °C	–	[44]
To analyze the metabolomic pathways and exercise in myocardial ischemia cases	Plasma	–	Sampling before, after, and 4 h after stress testing	Blood plasma preparation within 60 min; Storage at −80 °C	–	[81]
To assess the effect of physical exercise on the composition of metabolites in the blood	Plasma	EDTA tubes	10 h-fast; Sampling at rest, immediately after exercise, and 30 min later during 4 h of recovery	Centrifugation at 4000× *g* for 8 min at 0 °C; Storage at −80 °C	In the evening, subjects completed a cycle of intermittent, exhaustive cycling, followed by a low-carbohydrate meal.	[82]
To identify the network of metabolites regulating the beneficial effects of exercise	Plasma	–	Overnight fasting; Sampling at 0, 3, and 24 h after the end of training	–	First blood samples were taken at 8:15 a.m. Then, the subjects received a standardized breakfast (225 kcal). Samples were obtained after 1 h 45 min.	[29]
To analyze exercise-induced metabolic responses	Plasma	EDTA tubes	Sample collection before, at peak, and 60 min after exercise	Centrifugation at 2000× *g* for 10 min; Storage at −80 °C	–	[83]
To assess the impact of environmental heat stress during exercise	Serum	Lithium heparin tubes	–	Blood clotting at room temperature for 30 min; Centrifugation at 1500× *g* for 15 min at 4 °C; Storage at −80 °C	–	[84]
To identify the molecular signatures of exercise stress	Blood	Mitra^®^ device for volumetric absorborptive sampling	Collection of 20 µL of whole blood before and after graded exercise test and aerobic exercise	Drying of samples; Addition of a mixture of methanol:acetonitrile:water (5:3:2 *v*/*v*/*v*); Ultrasonic treatment for 1 h; Centrifugation at 18,000 × *g* for 10 min	–	[85]
To analyze the effects of respiratory muscle training at different exercise intensities on the metabolome profile	Serum	Serum separator tubes	12 h-fast	Blood clotting at room temperature (30 min); Centrifugation at 1450× *g* for 10 min; Storage at −80 °C	Blood samples were collected in the morning between 7:00 and 10:00. Participants were advised to avoid high-intensity exercise, stimulating foods and drinks, alcoholic beverages, and supplements on the day before blood collection.	[86]

EDTA—Ethylenediaminetetraacetic acid. * to limit possible daily effects on markers such as inflammation [87] and to avoid variation due to fasting state. ** as a means of reducing additional metabolic changes induced by the venesection stress as well as to limit interference to the athletes’ pre-marathon regimens.

**Table 4:** Collection and pre-treatment of urine samples for metabolite analysis.

Study Purpose	Tube Type	Conditions of Collection	Conditions of Sample Preparation and Storage	Explanation	Ref.
To analyze aerobic fitness level and metabolic profile	–	The urine samples were collected after a 12 h overnight fast.	Collection of the second urine sample; Centrifugation at 1800× *g* for 10 min at 4 °C; Storage of aliquots (1 mL) at −80 °C	–	[71]
To assess the effects of extreme physical exercise on the urine metabolome	–	–	Centrifugation; Storage of aliquots of at least 700 µL in presence of 0.05% NaN_3_ at −80 °C	–	[98]
To analyze metabolomic profiles on athletes’ urine after an 800 m race	–	The urine samples were collected before and after exercise.	Storage of urine samples at −80 °C	For two days, a standard diet consisting of carbohydrates, fats, and proteins provided 50%, 35%, and 15% of the daily calories; On the morning of the experimental day a standardized meal and 0.5 L of water during the morning meal, 1 L of purified water between the preexercise and post-exercise samplings	[99]
To explore urinary metabolome modifications after acute and chronic physical exercise	–	The urine samples were collected before and 30 min after each training session.	Storage of aliquots (10 mL) at −20 °C	Elapsed time since last voiding and volume of the spot urine check were carefully noted to calculate diuresis.	[100]
To analyze the changes in urine metabolome induced by two different exercise sessions	–	The urine samples were collected before and 35 min after training session.	Storage of aliquots at −80 °C	For two days, a standard diet consisting of carbohydrates, fats and proteins provided 50%, 35% and 15% of the daily calories *; On the morning of the experimental day a standardized meal and 0.5 L of water during the morning meal, 1 L of purified water between the preexercise and post-exercise samplings **	[46]
To perform urine metabolomic analysis for monitoring internal load in professional football players	–	First morning urine samples were collected after the rest day.	Storage of aliquots at −80 °C	All the players were advised to avoid strenuous physical activity during the rest day.	[101]
To assess the effect of high-intensity exercise on the urine metabolome	Urine collection jugs	The urine samples were collected 24 h before training session and during the following 24 h after training session.	Storage of aliquots (10 mL) in presence of 0.01% NaN_3_ at −80 °C	–	[102]
To analyze the effect of physical activity on urine metabolome	–	The urine samples were collected in the morning before breakfast.	Storage in presence of NaN_3_ at −20 °C	–	[103]
To assess the effect of physical activity on urine metabolome	Sterilized urine collection containers, Eppendorf tubes	The first urine samples were collected upon waking up, before and the next day after a submaximal training session.	Storage of urine samples in Eppendorf tubes at −80 °C; Storage of first (morning) and last (evening) samples at home in Eppendorf tubes at −20 °C.	Participants were instructed to refrain from consuming alcohol for 24 h and caffeine for 6 h before the test or consume anything other than water for 3 h before testing. Participants were asked not to exercise 24 h prior to the maximal test and for the duration of the urine collection period other than the submaximal trial.	[104]

* to reduce the impact of nutrient intake on metabolic profiling of the athletes. ** to diminish the effect of the hydration status on the urine production of athletes.

**Table 5:** Conditions for collection and pre-treatment of saliva samples for subsequent metabolite analysis.

Study Purpose	Conditions of Collection	Body Condition	Conditions of Sample Preparation and Storage	Explanation	Ref.
To study the saliva metabolome before and after a soccer match	Collection in polypropylene tubes (~2 mL)	Unstimulated whole saliva passive drooling before and after a soccer match	Centrifugation at 4000× *g* at 4 °C for 30 min; Aliquoting; Storage at −80 °C	–	[121]
To study the metabolite variations due to stress and fatigue in soccer athlete	Saliva samples were collected by 2 min chewing a polyester tampon, which was stored in the internal vial of the double-chambered tube.	–	Centrifugation at 1500× *g* for 15 min; Addition of NaN_3_; Storage at −20 °C	None of the athletes was using pharmaceutical or tobacco at the time of the study and all subjects had a controlled diet before the test.	[119]
To identify the salivary fatigue markers in soccer players	Saliva samples were obtained between 6:00 and 7:00 h in a quiet, temperature-controlled room.	–	Centrifugation at 1500× *g*; Storage at −80 °C	The subjects refrained from alcohol consumption and intense physical activity for 24 h prior to the measurements.	[122]
To study the effect of short-term intensive physical activity on the salivary metabolome	Sarstedt salivette polyester tube	The volunteers were asked not to brush their teeth within 1 h of collecting the samples.	Centrifugation at 1000× *g* for 20 min at 4 °C; Storage at −80 °C.	The collection of samples was carried out on 2 consecutive days, pre- and post-training sessions.	[123]
To study the impact of sleep quality on metabolite level and cognitive function in female volleyball athletes	Oral swab cotton swab and storage tube	Before the cognitive task test	Centrifugation at 1500× *g*; Storage at −80 °C	Participants were asked to abstain from any drink and food before the saliva collection.	[124]

**Table 6:** Conditions for collection and preprocessing of fecal samples for metabolite and microbiome analysis.

Study Purpose	Conditions of Collection	Body Condition	Conditions of Sample Preparation and Storage	Explanation	Ref.
To assess the impact of physical activity on the microbiome	Home stool collection kit	Participants were instructed to avoid alcohol for 3 days prior to stool collection	Samples were immediately stored in the participant’s freezer overnight and transported on ice to the lab; Samples were stored in −80 °C	–	[136]
To identify the characteristics of the gut microbiota in professional martial arts athletes	The fecal samples were self-collected in a polyethylene specimen collection system.	–	Samples were immediately placed on dry ice packs, and finally stored at −80 °C	Each athlete was asked to fill out the questionnaire on their frequency of consumption of food products over the past 3 months, their training durations and number of hours of exercise per week	[137]
To analyze the composition and function of gut microbiota in lean and obese individuals	Subjects were instructed to bring samples into the laboratory within 30 min of defecation *.	Fecal samples were collected at baseline, following six weeks of exercise, and following six weeks of return to sedentary activity.	Samples (~0.5 g) were aliquoted; Samples were stored at −80 °C	All collections were preceded by the 3-day food menu; Researchers asked participants the time defecation to ensure sample was received within 30 min	[138]
To analyze the effects of six weeks’ endurance exercise on the gut microbiota	–	Subjects self-collected the fecal samples before the control period, and after the control period and the exercise training period. The samples were always collected over 72 h after the last exercise bout.	Samples were frozen immediately at −20 °C after collection, brought to laboratory frozen and stored at −80 °C until processing	–	[139]
To assess the response of gut microbiota to metabolite changes induced by endurance exercise	Sterile sampling	Samples were collected before and after the half-marathon race.	Samples were immediately transported at 4 °C to the laboratory and stored at −80 °C until DNA and metabolite extraction	During the two sample time periods, each volunteer was given the same kind of food. Macronutrient intake information was obtained through a dietary questionnaire and calculated according to the China Food Composition.	[140]
To study the effect of acute moderate-intensity exercise on the fecal metabolomes	Participants were provided with a Fe–col stool collection device	The fecal samples were collected immediately before and after the workout **.	Samples were lyophilized using the Bench-Top freezedryer and stored at −80 °C.	–	[141]

* to ensure minimal degradation of volatile short-chain fatty acids. ** to avoid diet interference.

**Table 7:** Some methods for the preliminary preparation of plasma and serum samples for metabolite analysis.

Study Purpose	Method	Sample Type	Metabolite Extraction, Protein Precipitation	Conditions of Sample Preparation and Storage	Ref.
To study the effect of long-term physical activity on the serum metabolome	NMR	Serum	Methanol and dichloromethane	Slow thawing (overnight) in the refrigerator (+4 °C); Addition of sodium phosphate buffer (1:1 *v*/*v*); Metabolite extraction from the sample using a methanol-dichloromethane mixture and 0.15 M sodium chloride solution (1:25:25 *v*/*v*)	[70]
To analyze aerobic fitness level and metabolic profile	GC-MS	Plasma	Methanol	M etabolite extraction in methanol and water (1:8:1 *v*/*v*); Derivatization of the supernatant with methoxyamine	[71]
To study the relationship physical activity level and metabolic profile	LC-MS and GC-MS	Plasma	Methanol	Metabolite extraction in excess methanol	[73]
To study the association of diet, physical activity, cardiorespiratory fitness and obesity with serum metabolome	MS	Serum	Methanol	Addition of isotopically labeled internal standards; Derivatization with 5% phenyl isothiocyanate; Extraction in excess methanol	[74]
To study the metabolic response to acute hypoxic exercise and recovery	LC-MS	Serum	Methanol	Samples were prepared using the automated MicroLab STAR^®^ system; Extraction in excess methanol	[75]
To analyze the metabolic response to acute hypoxic exercise and recovery	LC-MS	Serum	Methanol	Metabolite extraction in serum, methanol, and water (1:8:1 *v*/*v*)	[43]
To analyze the effect of long-term physical activity on metabolic profile	LC-MS	Plasma	Acetonitrile	Metabolite extraction in acetonitrile (1:4 *v*/*v*); Isotopically labeled internal standard	[78]
To analyze the effect of long-term physical activity on metabolic profile	GC-MS	Serum	Mixture of chloroform and methanol	Extraction in a chloroform-methanol-water mixture (1:3:1 *v*/*v*); Protein precipitation with excess acetonitrile; Internal standard; Derivatization in BSTFA and TMCS	[79]
To study the metabolomic response after resistance exercise	NMR	Serum	–	Isotopically labeled internal standard	[80]
To assess the changes in plasma metabolome in response to cycling exercise	LC-MS	Plasma	Methanol	Metabolite extraction in excess methanol	[44]
To study the metabolomic pathways and exercise in myocardial ischemia cases	LC-MS	Plasma	Reverse-phase and normal-phase chromatography	Extraction of amino acids and amines were separated on a Luna phenyl-hexyl column by reverse-phase chromatography with an acetonitrile/water/0.1% acetic acid mixture at pH 3.5; Extraction of sugars and ribonucleotides were separated on a Luna amino column by normal-phase chromatography with an acetonitrile/water/0.25% ammonium hydroxide/10 mmol/L ammonium acetate mixture at pH 11; Extraction of organic acids were separated on a Synergi Polar-RP column by reverse-phase chromatography with an acetonitrile/water/5 mmol/L ammonium acetate mixture at pH 5.6 to 6.0	[81]
To study the effect of physical exercise on the composition of metabolites in the blood	NMR	Plasma	–	Analysis in heavy water (D_2_O)	[82]
To identify the network of metabolites regulating the beneficial effects of exercise	LC-MS	Plasma	Acetonitrile	Extraction in excess acetonitrile; Internal isotopically labeled standards	[29]
To study the exercise-induced metabolic responses	LC-MS	Plasma	Mixture of acetonitrile and methanol	Extraction in excess acetonitrile and methanol	[83]
To assess the impact of environmental heat stress during exercise	NMR	Serum		Thawing and diluting serum to 50% (*v*/*v*) with 10% NMR buffer (D_2_O with 100 mM sodium phosphate buffer at pH 7.4 and 0.1% azide), followed by centrifugation for 5 min at 21,500× *g* at 4 °C; 600 µL of the centrifuged sample is transferred to NMR tubes	[84]
To identify the molecular signatures of exercise stress	UHPLC-MS	Blood	Mixture of methanol and acetonitrile	Extraction in 9 volumes of an ice-cold ethanol-acetonitrile-water mixture (5:3:2, *v*/*v*); Isotopically labeled internal standards	[85]
To study the effects of respiratory muscle training at different exercise intensities on the metabolome profile	NRM	Serum	–	Isotopically labeled internal standards	[86]

BSTFA—bis(trimethylsilyl)-trifluoroacetamide; GC-MS—gas chromatography–mass spectrometry; LC-MS—liquid chromatography–mass spectrometry; MS—mass spectrometry; NMR—Nuclear Magnetic Resonance Spectroscopy; TMCS—trimethylchlorosilane; UHPLC MS—ultra-high-performance liquid chromatography–mass spectrometry.

**Table 8:** Some methods for the preliminary preparation of urine samples for metabolite analysis.

Study Purpose	Method	Metabolite Extraction	Conditions of Sample Preparation and Storage	Ref.
To analyze aerobic fitness level and metabolic profile	GC-MS	Methanol	Removal of urea, incubation in the presence of urease; Addition of internal isotopically labeled standard; Extraction of metabolites with excess methanol; Derivatization with methoxyamine;	[71]
To analyze the effects of extreme physical exercise on the urine metabolome	NMR	–	Centrifugation at 12,000× *g* for 5 min at 4 °C; Addition of urine buffer	[98]
To analyze the metabolomic profiles investigation on athletes’ urine after an 800 m race	NMR	–	Centrifugation at 12,000× *g* for 10 min at 4 °C	[99]
To explore the urinary metabolome modifications after acute and chronic physical exercise	NMR	–	Addition of buffer in D_2_O; centrifugation at 10,000× *g* for 5 min	[100]
To analyze the changes in urine metabolome induced by two different exercise sessions	NMR	–	Centrifugation at 1500× *g* for 5 min; Addition of buffer in D_2_O	[46]
To conduct the urine metabolomic analysis for monitoring the internal load in professional football players	UPLC-MS	–	Centrifugation at 10,000× *g* for 10 min at 4 °C; Addition of buffer; Addition of internal isotopically labeled standard	[101]
To analyze the effect of high-intensity exercise on the urine metabolome	NMR	–	Addition of buffer in D_2_O	[102]
To analyze the effect of physical exercise on the urine metabolome	NMR	–	Centrifugation at 13,000× *g* for 10 min; Addition of buffer in D_2_O	[103]
To analyze the effect of physical exercise on the urine metabolome	LC-MS	Acetonitrile	Addition of acetonitrile (1:4 *v*/*v*); Centrifugation	[104]

GC-MS—gas chromatography–mass spectrometry; LC-MS—liquid chromatography–mass spectrometry; NMR—Nuclear Magnetic Resonance Spectroscopy; UHPLC-MS—ultra-high-performance liquid chromatography–mass spectrometry.

**Table 9:** Some methods for the preliminary preparation of saliva samples for metabolite analysis.

Study Purpose	Method	Sample Preparation	Ref.
To study the saliva metabolome before and after a soccer match	NMR	Washing four times with 500 µL water and centrifugation at 13,800 g for 25 min at 4 °C; Ultrafiltration (3 kDa in 0.5 mL volume); Addition of D_2_O	[121]
To study the metabolite variations due to stress and fatigue in soccer athlete	NMR	Centrifugation at 4000 rpm, 4 °C for 1 h; Filtration using Amicon Ultra-4 Centrifugal filters membranes	[119]
To identify the salivary fatigue markers in soccer players	CE-TOFMS	Washing with water; Filter with a 5 kDa cut-off filter	[122]
To study the effect of short-term intensive physical activity on the salivary metabolome	LC-MS	Addition of isotopically labeled standard; Centrifugation at 8000 rpm for 10 min	[123]
To study the impact of sleep quality on metabolite level and cognitive function in female volleyball athletes	CE-TOFMS	Water rinse; Filtration with a 5 kDa cut-off filter	[124]

CE-TOFMS—capillary electrophoresis and time-of-flight mass spectrometry; LC-MS—liquid chromatography–mass spectrometry; NMR—Nuclear Magnetic Resonance Spectroscopy.

**Table 10:** Some methods of sample preparation for feces metabolites and microbiota analysis.

Study Purpose	Method	Conditions of Sample Preparation and Storage	Ref.
To assess the impact of physical activity on the microbiome	GC	50 mg of stool was homogenized with isopropyl alcohol, containing 2-ethylbutyric acid at 0.01% *v*/*v* as internal standard, at 30 Hz for 13 min using metal beads. Homogenates were centrifuged twice and supernatant was injected to Trace 1300 Gas Chromatograph, equipped with Flame-ionization detector.	[136]
To analyze the composition and function of gut microbiota in lean and obese individuals	GC	Fecal samples were acidified in 6.25% meta-phosphoric acid solution and stored at −20 °C. Acetic, n-butyric, and propionic acid solutions were used as standards	[138]
To analyze the response of gut microbiota to metabolite changes induced by endurance exercise	LC-MS	Feces (50 mg) were extracted with 800 μL methanol, and 10 μL DL-o-chlorophenylalanine (2.9 mg/mL) was then added as the internal standard. Samples were centrifuged at 12000 rpm and 4 °C for 15 min.	[140]
To assess the effect of acute moderate-intensity exercise on the fecal metabolomes	LC-HRMS	Samples were resuspended in 150 µL of water. 600 µL of methanol were added. Samples were lysed for 3 × 30 s at 6500 rpm and 4 °C;.Samples were left on ice for 90 min to facilitate complete protein precipitation.	[141]

GC-MS—gas chromatography–mass spectrometry; LC-HRMS—liquid chromatography coupled to high-resolution mass spectrometry; LC-MS—liquid chromatography–mass spectrometry.

**Table 11:** General storage conditions for biosamples until their shipping to the laboratory.

Item Used for Biosample Collection and Shipping	Storage Conditions	Acceptable Time for Shipping to the Laboratory	Ref.
Biosamples in a test tube with transport media	At room temperature (18–20 °C), in a dark place	Within 48 h	[171]
Sterile container with native biomaterial	At room temperature (18–20 °C), in a dark place	Within 2 h	
	At a temperature of 2–8 °C	Within 24 h	
	at a temperature of 37 °C	Within 2 h *	
Bottles with blood for sterility test	At room temperature (18–20 °C), in a dark place	Within 2 h	
		Within 24 h **	

*—Shipping of liquor, testing for meningococcus and pertussis. **—Delayed loading of bottles into the analyzer increases analysis time.

**Table 12:** Recommendations for arrangement of the storage area for biosamples until their shipping to the laboratory.

Storage Conditions	Biosample Storage Area	Ref.
At 18–20 °C, in a dark place	Closed refrigerated cabinet, box away from heating devices	[172]
	Closeable container for shipping	
At 2–8 °C	Refrigerator	
	Thermal container with ice packs	
At 37 °C	Thermostat	
	Container with ice packs maintained at desired temperature (heating pad, wrap with a disposable diaper or a rag)	

## Data Availability

The data compiled for this review paper are publicly available and can be found in the listed “References”.

## Supplementary Materials

The following supporting information can be downloaded at: https://www.mdpi.com/article/10.3390/biom14121561/s1, Table S1: Assessment of study participant lifestyle.
