# Supplementary material for: Sample Preparation for Metabolomic Analysis in Exercise Physiology
Source: Biomolecules. 2024 Dec 7;14(12):1561. doi: 10.3390/biom14121561 (PMC11673972; doi:10.3390/biom14121561)
Supplement: Supplementary file 1 [file biomolecules-14-01561-s001.zip › biomolecules-3267445-supplementary.pdf]

Table S1. Assessment of study participant lifestyle

**General data**	
Gender	female/male
Age, years	
Height, cm	
Weight, kg	
BMI, kg/m^2	
**General assessment of the level of physical activity**	
How often are you engaged in physical activity per week?	◯ not engaged;<br>◯ engaged 1 to 2 times a week;<br>◯ engaged 3 to 4 times a week;<br>◯ engaged 5 to 6 times a week;<br>◯ engaged every day
What is the duration of your physical activity?	◯ up to 30 min.; ◯ up to 1 h; ◯ 1 to 2 h;<br>◯ more than 2 h
How intense is your physical activity?	◯ light;<br>◯ moderate;<br>◯ heavy
How many minutes do you walk a day?	◯ up to 30 min; ◯ up to 1 h;<br>◯1 to 2 h; ◯more than 2 h
How many hours a day do you spend in a sitting position?	◯ up to 2 h; ◯ up to 5 h;<br>◯ 5–8 h; ◯ more than 8 h
How do you spend your free time on weekends?	____________________<br>____________________<br>____________________
**Assessment of the latest physical activity**	
Latest physical activity	Date: __:__:____; Time: __:__
Sport	____________________<br>____________________
Type of physical activity	◯ aerobic activity;<br>◯ anaerobic activity;<br>◯ mixed activity
Intensity of physical activity	◯ light; ◯ moderate; ◯ heavy
Duration of physical activity	____________________<br>____________________
**General diet characteristics**	
Average number of meals per day	◯ 2; ◯ 3; ◯ more than 4
Do you eat fruits and vegetables every day?	◯ Yes; ◯ No
**General characteristics of the nutrition mode**	
Do you eat at the same time every day?	◯ Yes; ◯ No
How often do you eat fast food?	◯ never; ◯ 1 a month;<br>◯ 1 a week; ◯ 2x a week;<br>◯ more than 2 times per week

Last meal characteristics	
Last meal	Date: __:__:____; Time: __:__
Last meal composition	____________________<br>____________________<br>____________________<br>____________________
Do you feel a sense of fullness after eating?	◯ Yes; ◯ No
**Stress level assessment**	
Are you experiencing stress at the moment?	◯ Yes; ◯ No
How often do you feel stress?	◯ Yes; ◯ No
Is it easy to put you out of temper?	◯ Yes; ◯ No
Are you susceptible to stress?	◯ Yes; ◯ No
Have you felt stress in the last week?	◯ Yes; ◯ No
**Additional information**	
Do you take any drugs?	◯ Yes; ◯ No<br>____________________<br>____________________
Do you use any nutritional supplements?	◯ Yes; ◯ No<br>____________________<br>____________________
